# Development of Practical Low-Volume Screening Method and Pharmacokinetic Simulation of Levofloxacin-Loaded Nanofiber Inserts for Sustained Ocular Therapy

**DOI:** 10.3390/pharmaceutics17101343

**Published:** 2025-10-17

**Authors:** Houssam Aaref Abboud, Romána Zelkó, Adrienn Kazsoki

**Affiliations:** University Pharmacy Department of Pharmacy Administration, Semmelweis University, Hőgyes Endre Street 7-9, 1092 Budapest, Hungary; houssam.abboud@phd.semmelweis.hu

**Keywords:** levofloxacin, nanofibrous inserts, ophthalmic drug delivery, small-volume dissolution, pharmacokinetic modeling, in vitro antibacterial study, sustained release

## Abstract

**Background**/**Objectives**: Ocular drug delivery faces significant challenges due to anatomical and physiological barriers that limit drug bioavailability, particularly with conventional eye drops. Levofloxacin (LEVO), a broad-spectrum antibiotic, is widely used in the treatment of bacterial conjunctivitis, but its therapeutic efficacy is hindered by rapid precorneal clearance and short residence time. **Methods**: This study introduces a biorelevant 2 mL dissolution model to simulate ocular conditions better and evaluate the release kinetics of LEVO-loaded nanofibrous ophthalmic inserts. Compared to the conventional 40 mL setup, the 2 mL system demonstrated a slower and more sustained drug release profile, with kinetic modeling confirming a more controlled release behavior. Difference and similarity factor analysis further validated the distinct release profiles, highlighting the impact of dissolution volume on release dynamics. **Results**: Preliminary pharmacokinetic modeling suggested that the nanofiber inserts, particularly when applied twice daily, maintained levofloxacin concentrations above minimum inhibitory and bactericidal levels for extended durations across three bacterial strains (*Escherichia coli*, *Pseudomonas aeruginosa*, and *Staphylococcus aureus*), potentially outperforming traditional eye drops. **Conclusions**: These findings suggest that small-volume dissolution testing may provide a more realistic method for evaluating ophthalmic insert formulations, though in vivo validation is needed. Moreover, the nanofibrous inserts show potential as a sustained-release alternative that warrants further investigation to improve patient compliance and therapeutic outcomes in ocular disease management.

## 1. Introduction

Delivering drugs to the eye remains one of the most formidable challenges in pharmaceutical research because anatomical and physiological barriers dramatically reduce drug availability at ocular tissues [1]. Conventional ophthalmic preparations, such as eye drops and gels, typically achieve less than 5% bioavailability [2,3,4]. This poor efficiency is largely attributed to precorneal factors, including tear dilution, reflex blinking, rapid tear turnover, and nasolacrimal drainage, all of which contribute to the rapid elimination of the drug from the ocular surface [5]. Consequently, these formulations often require frequent administration to maintain therapeutic levels, which can lead to poor patient compliance and suboptimal clinical outcomes [6]. To address these limitations, solid ocular dosage forms—particularly inserts placed in the conjunctival sac—have emerged as promising alternatives [7,8,9]. These inserts are designed to provide prolonged residence time, controlled drug release, and improved bioavailability. Among the various types, nanofibrous ocular inserts fabricated by electrospinning have gained significant attention in recent years [10,11,12,13]. Electrospinning is a versatile and scalable technique that produces ultra-fine fibers, typically in the nanometer range, resulting in a large surface-area-to-volume ratio, high porosity, and tunable mechanical and physicochemical properties [14,15,16,17,18,19,20,21]. These features allow for efficient drug loading, sustained release profiles, and enhanced adhesion to the ocular surface, even in the dynamic environment of the eye [18].

Nanofibrous inserts can be engineered from biodegradable and biocompatible polymers, such as polyvinyl alcohol (PVA) [22,23], chitosan [23], cellulose derivatives [24], or polycaprolactone [25,26], making them suitable for ophthalmic applications [22,23,24,27,28,29,30]. Furthermore, these systems can be functionalized with permeation enhancers, mucoadhesive agents, or stimuli-responsive components to further enhance ocular drug delivery [30,31,32,33]. In addition to overcoming the barriers associated with conventional formulations, electrospun inserts offer the potential for reduced dosing frequency, minimized systemic absorption, and better therapeutic efficacy [32].

Thus, the development of nanofibrous ocular inserts via electrospinning presents a promising strategy to revolutionize ocular drug delivery, offering a controlled, patient-friendly, and efficient alternative to traditional topical applications.

In our previous work, we developed preservative-free nanofiber-based ophthalmic inserts loaded with levofloxacin (LEVO), demonstrating improved drug stability and promising antimicrobial activity [34]. A key assessment in that study was a dissolution test using 40 mL of phosphate-buffer solution. Although dissolution volumes ranging from 25 to 40 mL have been widely used in previous in vitro release studies for ocular formulations [35,36,37,38], including those utilizing dialysis bag diffusion systems, these relatively large volumes poorly reflect the real physiological conditions of the eye, where tear volume is only 7–8 µL and the conjunctival sac can hold 15–30 µL [5]. Large-volume dissolution tests may overestimate release rates and fail to reveal key formulation features, such as the gel-like transition of nanofiber inserts that act as an additional barrier to diffusion. Using a smaller 2 mL volume highlighted this transition and the inserts’ sustained-release capability, offering a clearer perspective on formulation performance while acknowledging that neither setup fully captures tear film dynamics.

Conventional static immersion methods, such as placing an insert in 20–50 mL of buffer with gentle agitation, offer simplicity and convenient sampling but suffer from significant over-dilution. In these large-volume baths, the concentration gradient driving drug diffusion is artificially elevated, leading to faster release profiles that may misrepresent actual ocular release conditions [39,40,41]. Franz diffusion cells and other membrane-based assays introduce a barrier to mimic corneal permeability and remain easy to implement, but they also rely on receptor compartments of tens of milliliters and cannot reproduce rapid tear clearance or low fluid turnover [23,42,43,44,45]. Flow-through systems, such as USP Apparatus 4, improve upon static designs by continuously renewing the medium and controlling hydrodynamics; however, they demand complex instrumentation, larger reservoirs, and careful validation to ensure flow rates do not exaggerate release kinetics [40,42,45,46].

More recently, miniaturized static approaches using 2 mL chambers have been proposed to bridge this gap. By reducing the dissolution medium to a few milliliters, these small-volume devices more closely approximate tear-film volume, conserve expensive simulated tear fluid components and active pharmaceutical ingredients, and enhance analytical sensitivity—small increments of drug release produce larger concentration changes detectable by HPLC or UV–Vis. At the same time, a well-engineered design is required to maintain sink conditions and avoid excessive shear at such small scales. In summary, each method balances simplicity against physiological relevance, but none fully recreates the complex, low-volume, high-turnover environment of the tear film.

Therefore, we introduce a 2 mL static dissolution model—chosen to approximate tear volume more closely, minimize medium and API consumption, and increase analytical sensitivity—to compare LEVO release from nanofibrous inserts versus standard eye drops. By evaluating release profiles and conducting preliminary pharmacokinetic modeling against three bacterial strains, we aim to assess the potential prolonged release effect and therapeutic potential of the nanofibrous system, while proposing a more predictive in vitro–in vivo correlation platform for ophthalmic drug delivery research. This work represents an initial step toward developing more biorelevant testing methods, though comprehensive validation studies will be necessary to establish clinical relevance.

## 2. Materials and Methods

### 2.1. Materials

Levofloxacin (LEVO) was sourced from Merck Ltd. (Budapest, Hungary), as were the polymers polyvinyl alcohol (PVA, Mowiol^®^ 18–88, average Mw ~130 kDa) and Poloxamer (average Mw ~12.6 kDa). Hydroxypropyl-β-cyclodextrin (HP-β-CD, degree of substitution ~4.5) was provided by Cyclolab Ltd. (Budapest, Hungary), while Richter Gedeon Plc kindly supplied sodium hyaluronate (Budapest, Hungary). Potassium dihydrogen phosphate, sodium hydroxide, and ethylenediaminetetraacetic acid were obtained from Molar Chemicals Ltd. (Budapest, Hungary). Pharmaceutical-grade distilled water was used for all solution preparations.

### 2.2. Precursor Solution Preparation for the Electrospinning Process

Based on our previously published work, the polymer matrices were composed of PVA and Poloxamer in an 8:2 mass ratio, with a total polymer concentration of 12% (*w*/*w*). First, PVA was dissolved in water by stirring at 80 °C until a clear solution formed. After cooling to room temperature, Poloxamer was added and stirred until fully homogenized. LEVO was incorporated at 3% (*w*/*w*), and its solubility was enhanced by including HP-β-CD in a 1:1 molar ratio with LEVO. Finally, sodium hyaluronate was added at a concentration of 0.2% (*w*/*w*) and stirred until the mixture was uniform [12].

### 2.3. Preparation of Nanofibers

Nanofibrous samples were prepared using a laboratory-scale electrospinning apparatus (SpinCube, SpinSplit Ltd., Budapest, Hungary). Precursor solutions were loaded into 1 mL plastic syringes and connected via tubing to a 22 G needle. Each syringe was mounted on a pump to maintain a continuous flow of solution. Aluminum foil or baking paper was stuck onto the collector to collect each sample for further analysis. The process was performed at ambient conditions of 22 ± 1 °C temperature and 40 ± 5% relative humidity. During the fiber formation, 0.15 µL/s flow rate, 22.7 ± 0.5 kV was used, and the collector–emitter distance was kept at 12.5 cm [12].

### 2.4. Small-Volume Dissolution Study

The dissolution behavior of levofloxacin-loaded nanofibrous ophthalmic inserts was first characterized using the 40 mL setup we described in our previous publication (pH 7.4 phosphate buffer, magnetic stirring at 200 rpm). An in-line UV–Vis probe (Jasco V-750, ABL&E-JASCO Magyarország Ltd., Budapest, Hungary) recorded absorbance at a wavelength of 290 nm every 10 s to monitor drug release continuously [12].

**Figure 1:**
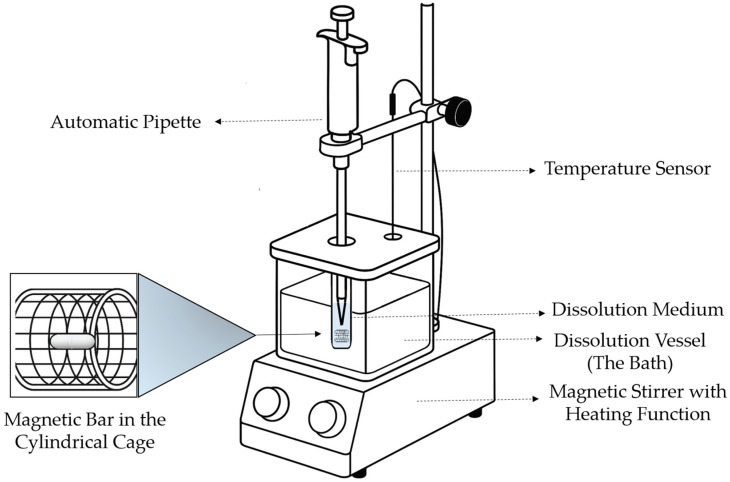
Schematic representation of the custom-made small-volume dissolution device.

Building on that work, we then developed a custom small-volume (2 mL) dissolution device, also using pH 7.4 phosphate buffer, to better approximate ocular conditions. Figure 1 schematically illustrates the developed dissolution device.

To evaluate the in vitro drug release from the test samples, a custom-designed small-volume dissolution testing apparatus was developed. The system integrates temperature control, magnetic stirring, and manual sampling through a compact and reproducible configuration tailored for nanoformulations or low-dose delivery systems. The core components of the setup are as follows:

- Dissolution Vessel: A square-shaped borosilicate glass container (8 cm × 8 cm base, 6 cm height) was used as the dissolution bath. It was filled with water and maintained at the desired temperature throughout the experiments. To minimize evaporation of the dissolution medium, a custom-designed lid was 3D-printed using polylactic acid (PLA) filament (basic black PLA, SNAPMAKER HK LIMITED, Hong Kong, China). The lid was fabricated with a Snapmaker 2.0 Modular 3-in-1 3D Printer A350T (SNAPMAKER HK LIMITED, Hong Kong, China).

A tightly fitting lid made of chemically resistant plastic was fabricated to cover the vessel and minimize evaporation. Two precisely drilled apertures were included: a central sampling port allowed vertical insertion of the automatic pipette, and a rear-side port accommodated the temperature probe, ensuring immersion in the dissolution medium without disturbing the stirring process.

The thread was threaded into the lid, into which the screw-mouth glass jar (inner diameter: 1 cm, length: 4 cm) fitted snugly.

- Magnetic Stirrer with Heating Function: An IKA RCT Basic safety control stirrer (IKA-Werke GmbH & Co. KG, Staufen im Breisgau, Germany) served as the base. The device featured two control knobs for independent regulation of temperature and stirring speed. The heating plate ensured homogeneous thermal distribution throughout the medium.
- 3D printed custom cylindrical cage: The predetermined size and weight nanofibrous sheets were screwed on a magnetic stirring bar (diameter: 2 mm and length: 7 mm) and it was placed into a 3D printed custom cylindrical cage (inner diameter: 0.5 cm, length: 1 cm), which ensured that the nanofibrous sample was immersed into the dissolution medium.
- Automatic Pipette Assembly: The pipette was vertically mounted onto an external retort stand, aligned with the center of the vessel for reproducible sample withdrawal. In this model, 200 µL aliquots were withdrawn at predetermined intervals.
- Temperature Sensor: A digital probe was affixed to a vertical rod via a clamp. The probe extended through the designated port on the vessel lid to monitor the temperature within the dissolution vessel (water bath), ensuring consistent thermal control of the medium (±0.2 °C).

In the experimental setup, the levofloxacin-loaded insert was carefully wrapped around the magnetic bar and placed inside a cylindrical cage positioned into the vial in the dissolution bath on the heater plate. The dissolution medium was then directly poured into the same vial containing the sample, allowing continuous stirring and uniform contact between the insert and the medium throughout the test.

This integrated system enabled controlled and repeatable in vitro dissolution testing of sensitive and miniaturized drug delivery systems such as nanofibrous inserts. The design offers flexibility, low-cost implementation, and compatibility with analytical follow-up using spectrophotometric or chromatographic techniques.

During the measurements, 200 µL aliquots were withdrawn at predetermined intervals–no shorter than 30 s between measurements–and immediately replaced with fresh buffer to maintain sink conditions. Each dissolution experiment was performed in triplicate using independent fiber samples, and the aliquots were analyzed in a 0.5 cm quartz cuvette at 290 nm on the same Jasco V-750 spectrophotometer.

#### Data Analysis and Limitations

Because the 40 mL and 2 mL experiments employed different sampling methods and time resolutions, we first aligned both datasets by structuring a unified time-point matrix. Drug release profiles from each setup were then fitted to the Weibull model, which is well established for characterizing controlled-release kinetics. However, we acknowledge that the Weibull model represents a simplified approximation of the complex release mechanism involved. The Weibull equation is expressed as:

(1) $$y=A\times \left(1-exp\left(-{\left(\frac{x-x_{c}}{k}\right)}^{d}\right)\right)$$

where

*y* = cumulative percentage of drug released (%),
*A* = asymptotic maximum dissolution (%),
*x* = time (sec),
*x_c_* = lag time (sec),
*d* = shape factor (controls the release kinetics),
*k* = scale parameter (related to the time required for drug release).

The fitting procedure was performed using nonlinear regression analysis in Python 3.12, employing the SciPy optimization function. Initial parameter estimates were refined based on experimental observations to achieve optimal convergence.

Two model-independent parameters, the difference factor (*f*_1_) and the similarity factor (*f*_2_), were calculated to compare the dissolution profiles obtained from 2 mL and 40 mL dissolution media.

Difference factor (*f*_1_):

(2) $$f_{1}=\sum \left|R_{t}-T_{t}\right|\times 100/\sum R_{t}$$

where

*R_t_* is the percentage of drug released at time t from the reference (40 mL);
*T_t_* is the percentage of drug released at time t from the test sample (2 mL)

Similarity factor (*f*_2_):

(3) $$f_{2}=50\times \log((1+\frac{1}{n}\sum \left(R_{t}-T_{t}{{)}^{2})}^{-0.5}\times 100\right)$$

where

*n* is the number of time points.

### 2.5. Pharmacokinetic Model

We developed a preliminary pharmacokinetic model to compare the pharmacokinetics of LEVO delivered via traditional eye drops and a 10 mg nanofiber insert. This comparison incorporates the effects of blinking and enhanced permeability on drug retention and release, focusing on maintaining therapeutic levels against specific pathogens.

#### 2.5.1. Key Parameters and Assumptions

It is important to note that the following parameters are based on literature values and theoretical considerations.

##### Eye Drop Model

- Drug concentration and dosing: 5 mg/mL, applied in every 2 h.
- Dose per drop: 0.25 mg per application (0.05 mL volume/drop).
- Dosing: 12 doses per day over 24 h.
- Determination of clearance rate for eyedrops:

The ocular clearance of topically applied drugs is a key limiting factor in achieving and maintaining therapeutic concentrations on the eye surface. Rapid elimination occurs via tear turnover, nasolacrimal drainage, and reflex blinking, significantly reducing precorneal drug residence time.

Several studies have reported short precorneal half-lives for ophthalmic solutions. Schoenwald highlighted that the half-life of most topically applied drugs in the tear film ranges from 1 to 4 min due to physiological protective mechanisms such as blinking and drainage [47]. Similarly, Urtti, in his article, emphasized the limitations posed by ocular barriers and fluid dynamics, reporting that over 90% of an instilled drug dose may be lost within the first few minutes [48]. Barar et al. also noted that pre-corneal fluid drainage and blinking contribute to rapid drug clearance, severely limiting ocular bioavailability [49]. Despite these short residence times, studies examining tear pharmacokinetics of levofloxacin showed that drug concentrations in the tear film remain measurable for hours due to high initial dosing and formulation retention [50]. In their study, levofloxacin concentrations peaked at 221 µg/mL at 15 min and decreased to 17 µg/mL at 4 h, corresponding to an estimated terminal elimination half-life of ~1 h. However, this prolonged terminal phase reflects residual drug in the tear meniscus and does not accurately represent the initial rapid clearance.

Therefore, to conservatively model the initial rapid elimination phase, a half-life of 10 min (0.167 h) was selected in this simulation. This value represents a midpoint between literature-reported tear clearance (1–4 min) and the slower terminal elimination phase and aligns with pharmacokinetic modeling practices that emphasize early-phase drug loss after drop instillation. The corresponding first-order clearance rate constantly used in the model was:

(4) $$k_{clear\_eye\;drops}=\frac{ln(2)}{t_{1}}=\frac{0.693}{0.167\;h}\sim 4.15\;h^{-1}$$

##### Nanofibrous Insert Model

- Insert Weight: 10 mg, levofloxacin at 10% (*w*/*w*).
- Drug Content per Insert: 1 mg.
- Drug Release Rate Constant (*k_release_*).

The in vitro dissolution profile of the nanofibrous insert was evaluated in 2 mL of simulated tear fluid. Approximately 90% of levofloxacin was released within 300 s (5 min). Assuming first-order release kinetics, the release rate constant was calculated using the standard exponential release model:

(5) $$k_{release}=-\frac{1}{t}\times ln\left(1-\frac{c(t)}{c_{\infty }}\right)$$

Substituting:

$$\begin{array}{c} t=5\min=0.0833h\\ \frac{c(t)}{c_{\infty }}=0.9\\ k_{release}=\frac{2.3026}{0.0833}\sim 27.64\;h^{-1} \end{array}$$

This value reflects the rapid initial drug release from the electrospun nanofiber matrix under low-volume, tear-like conditions.

- Ocular Clearance rate:

Compared to conventional eye drops, the nanofibrous insert demonstrates enhanced retention on the ocular surface. This is attributed to:

- Hydration-triggered in situ gelation,
- Mucoadhesion of the nanofiber matrix,
- Reduced exposure to nasolacrimal drainage.

Multiple studies on mucoadhesive and gel-forming systems have reported prolonged ocular residence times, with half-lives ranging from 4 to 12 h, depending on formulation characteristics [51,52,53]. For this simulation, a conservative half-life of 7 h was assumed, resulting in:

(6) $$k_{clear\_\;insert}=\frac{ln(2)}{7h}\sim 0.099\;h^{-1}$$

This value appropriately reflects slower drug elimination compared to eye drops (which typically exhibit clearance rates > 4 h^−1^) and is consistent with literature reports for in situ gelling and nanofiber-based systems.

- Dosing: Both once-daily and twice-daily applications were simulated to determine their efficacy.

##### Pathogen-Specific Minimum Inhibitory Concentrations (MIC) and Minimum Bactericidal Concentration (MBC) Thresholds

To evaluate the efficacy of the nanofibrous formulation against specific pathogens, MIC and MBC thresholds were set as follows (Table 1):

#### 2.5.2. Model Equations

The following equations represent simplified approximations of complex ocular pharmacokinetics.

##### Eye Drop Concentration Model


(7) $$c_{eye\;drop}(t)=\sum_{n=0}^{12}{(0.025\times e}^{-k_{clear_{eye_{drop}}}\times \left(t-n2\right)})\;\;\;for\;t\geq n\times 2h$$

##### Nanofiber Insert Concentration Model for a Single Application


(8) $$c_{insert}(t)=\frac{C_{insert}}{k_{release}}\times (1-e^{-k_{release}\times t})\times e^{-k_{clear}\times t}$$

##### Nanofiber Insert Concentration Model for Twice-Daily Application


(9) $$c_{insert}\left(t\right)=c_{insert}\left(t\right)+c_{insert}\left(t-12\right)\;\;\;for\;t\geq 12h$$

This method provides a detailed framework for comparing the pharmacokinetic profiles of the eye drop and nanofiber insert formulations, with attention to pathogen-specific MIC levels and dosing efficacy.

## 3. Results

### 3.1. Small Volume Dissolution

**Figure 2:**
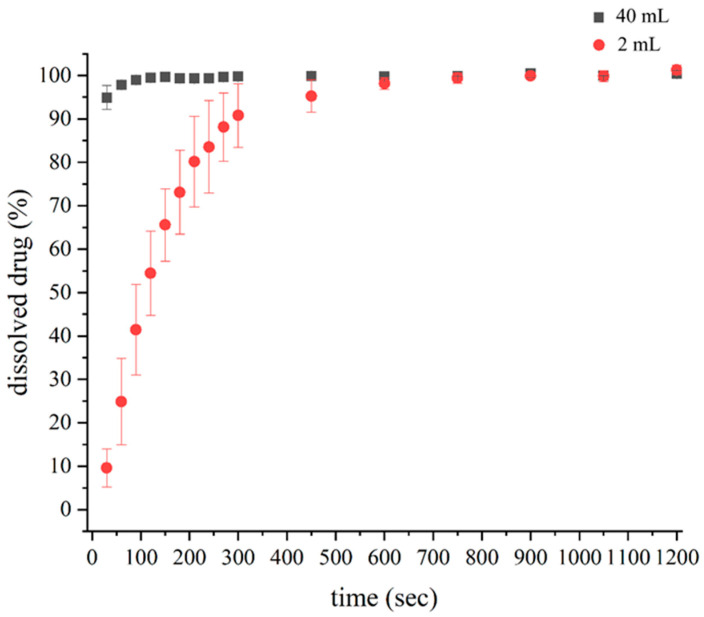
Dissolution profile of the levofloxacin-loaded nanofibrous ophthalmic insert performed in 2 mL and 40 mL phosphate buffer (pH = 7.4) (The data are presented as mean ± SD (*n* = 3).).

The in vitro dissolution behavior of levofloxacin-loaded nanofibrous ophthalmic inserts was investigated using two media volumes: 2 mL and 40 mL (which was obtained in our previous work [12]) of phosphate buffer (pH 7.4). The resulting drug release profiles are depicted in Figure 2.

To quantitatively characterize the dissolution kinetics, the data were fitted to the Weibull model. The fitted parameters are presented in Table 2, highlighting the substantial differences in drug release behavior under the two testing conditions.

The difference and similarity factors were calculated to quantify the difference between the two dissolution profiles. The results showed that the *f*_1_ was 27.74 while the (*f*_2_) was 21.37. For the similarity, *f*_1_ should be <15, and *f*_2_ should be >50.

### 3.2. Pharmacokinetic Simulation

**Figure 3:**
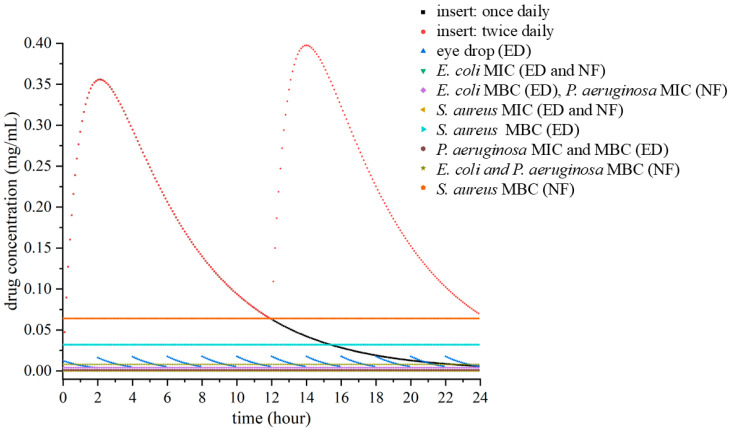
Simulated levofloxacin concentration–time profiles in tear fluid following administration of a nanofiber insert once daily (black solid line, ■ markers), nanofiber insert twice daily (red solid line, ● markers), and eye drops applied every 2 h (blue solid line, ▲ markers). Horizontal dashed lines represent pathogen-specific antibacterial thresholds: *E. coli* MIC (green), *E. coli* MBC (cyan), *S. aureus* MIC (purple), *S. aureus* MBC (orange), *P. aeruginosa* MIC (turquoise), and *P. aeruginosa* MBC (brown). Solid horizontal lines correspond to values determined for both nanofibers (NF) and eye drops (ED), as indicated in the legend.

Pharmacokinetic modeling was performed to simulate the ocular drug concentration-time profiles of levofloxacin following three different administration strategies:

(i) a once-daily application of the nanofibrous insert,
(ii) a twice-daily insert regimen, and
(iii) conventional eye drops administered every two hours. The simulated profiles are shown in Figure 3.

All relevant MIC and MBC values, specific to both the nanofibrous formulation and the eye drops, were overlaid on the figure as color-coded horizontal reference lines. This approach enabled a direct, visual assessment of each dosing regimen’s ability to achieve and sustain therapeutic drug levels relative to pathogen-specific pharmacodynamic targets.

## 4. Discussion

The 40 mL medium exhibited a rapid drug release, with nearly complete dissolution occurring within the first few minutes. In contrast, the 2 mL dissolution medium demonstrated a bit prolonged drug release, highlighting the impact of a reduced medium volume on the dissolution kinetics. The slower drug release observed in the 2 mL system suggests that the in situ gelation effect was more pronounced, potentially leading to prolonged ocular drug retention.

The observed differences in drug release between the 2 mL and 40 mL dissolution media can be explained by the limited solubility of levofloxacin (LEVO) at pH 7.4. Each ocular insert contained 1 mg of LEVO, and in the small-volume (2 mL) setup, the drug concentration in the medium may approach its solubility limit (~54.2 µg/mL). Under these conditions, saturation effects play a crucial role, reducing the concentration gradient and slowing drug diffusion from the insert. In contrast, the larger 40 mL medium maintains sink conditions and a higher concentration gradient, promoting faster drug release. This solubility-limited behavior highlights the importance of considering medium volume in dissolution studies, as it can significantly influence release profiles and, consequently, the potential in vivo performance of ocular formulations.

To quantitatively describe these differences, the release data were fitted to the Weibull model, which revealed distinct dissolution behaviors under the two testing conditions (Table 2). Notably, the shape factor (d) was substantially higher in the 2 mL system (0.915) compared to the 40 mL system (0.159), indicating that drug release under low-volume conditions is more diffusion-controlled. In the 2 mL environment, limited dilution and higher local drug concentrations likely promote matrix swelling and gel formation, creating a diffusional barrier that slows release. Conversely, the larger 40 mL volume facilitates rapid diffusion due to the availability of excess solvent, minimizing the impact of gelation and leading to faster drug release.

These findings suggest that under practical low volume screening conditions, the nanofiber matrix behaves more like a sustained-release depot, offering prolonged drug release more representative of in vivo performance. The Weibull model parameters, and the marked differences in similarity (*f*_2_ = 21.37) and difference (*f*_1_ = 27.74) factors further support that the 2 mL setup provides a more physiologically relevant in vitro model for ocular drug delivery assessment.

The simulated pharmacokinetic profiles were then analyzed in relation to pathogen-specific minimum inhibitory concentration (MIC) and minimum bactericidal concentration (MBC) values for *E. coli*, *S. aureus*, and *P. aeruginosa*, as shown in Table 1. Notably, these thresholds differ between the commercial eye drop and the nanofibrous insert, reflecting distinct formulation attributes such as solubility, release behavior, and ocular surface interaction.

The twice-daily administration of the nanofibrous insert produced two sustained concentration peaks, maintaining drug levels above both the MIC and MBC thresholds for *E. coli* and *P. aeruginosa* associated with the insert. Although the MBC for *S. aureus* was not fully achieved, concentrations remained above the MIC for a considerable duration, suggesting potential therapeutic benefit through bacterial growth suppression. These results demonstrate the superior consistency and prolonged antimicrobial coverage provided by the twice-daily insert regimen, particularly for moderately resistant pathogens.

In contrast, the pharmacokinetic profile of the conventional eye drops revealed sharp, short-lived spikes in drug concentration that fell below the MIC and MBC values for most of the dosing interval. Only transient exceedance of the *E. coli* MIC was observed, with overall poor alignment to bactericidal targets. This reflects the inherent limitations of eye drops, primarily due to rapid precorneal clearance and minimal retention on the ocular surface.

Taken together, these results confirm that nanofibrous ophthalmic inserts—especially when used twice daily—offer significantly improved pharmacokinetic profiles and more reliable attainment of antimicrobial thresholds compared to traditional eye drops. The ability of the insert to sustain drug levels near or above formulation-specific MBCs for extended periods is critical for achieving effective bacterial eradication. Moreover, the differing MIC and MBC values between formulations highlight the importance of assessing therapeutic performance within the context of each delivery system’s unique bioactivity profile.

Although the employed 2 mL dissolution medium is still considerably larger than the physiological tear volume (8–10 μL), it represents better condition compared to the conventional 40 mL setup. Importantly, the reduced medium volume enabled clear observation of the transition of the nanofibrous inserts into a gel-like state, a phenomenon that acts as an additional physical barrier to drug diffusion. This gelation behavior, which was masked under the 40 mL condition, highlighted the inherent ability of the nanofiber matrices to sustain drug release. Therefore, while the 2 mL system does not fully replicate the in vivo ocular environment, it provides a valuable balance between experimental feasibility and physiological relevance, offering deeper insight into the performance of the developed formulations.

## 5. Conclusions

This study proposes the value of small-volume dissolution testing and pharmacokinetic modeling as powerful tools in the biorelevant assessment of ophthalmic drug delivery systems. The 2 mL dissolution model more accurately reflects tear film conditions, offering a controlled and prolonged levofloxacin release profile from nanofibrous inserts compared to conventional 40 mL setups. The distinct release kinetics and model-independent parameters confirm that reducing the dissolution volume leads to more predictive in vitro behavior aligned with ocular physiology.

Pharmacokinetic simulations further demonstrated that nanofibrous inserts, particularly when applied twice daily, sustain levofloxacin concentrations above pathogen-specific MIC and MBC values for extended durations. This enhanced therapeutic coverage notably surpassed the performance of traditional eye drops, which showed transient peaks and subtherapeutic troughs due to rapid precorneal clearance.

Together, these findings support the potential of levofloxacin-loaded nanofibrous ophthalmic inserts as a promising alternative to conventional topical formulations. The integration of more biorelevant in vitro testing with pharmacodynamic-informed modeling represents a promising approach for optimizing ophthalmic drug delivery, though substantial validation work is required to realize its full potential. These strategies may ultimately contribute to the development of more effective, patient-friendly treatments for bacterial conjunctivitis and other ocular infections.

Despite its limitations, this work contributes valuable insights to the field by highlighting the importance of biorelevant testing conditions for specialized delivery systems, demonstrating the potential of integrated dissolution–pharmacokinetic modeling approaches, and providing a framework for future method development and validation studies. The small-volume dissolution approach may represent a step toward more physiologically relevant testing, though comprehensive validation is needed.

## Figures and Tables

**Table 1:** Minimal Inhibitory Concentration (MIC) and Minimal Bactericidal Concentration (MBC) values of commercially available eye drop and levofloxacin-containing nanofibrous formulation against *Escherichia coli* (*E. coli*), *Staphylococcus aureus* (*S. aureus*), and *Pseudomonas aeruginosa* (*P. aeruginosa*) [43]. Minimal Inhibitory Concentration (MIC) and Minimal Bactericidal Concentration (MBC) values of commercially available eye drop and levofloxacin-containing nanofibrous formulation against *Escherichia coli* (*E. coli*), *Staphylococcus aureus* (*S. aureus*), and *Pseudomonas aeruginosa* (*S. aeruginosa*) [12].

Pathogen	Nanofibrous Sample		Eye Drop	
	MIC (µg/mL)	MBC (µg/mL)	MIC (µg/mL)	MBC (µg/mL)
*E. coli*	<0.125	8	<0.125	4
*S. aureus*	0.25	64	0.25	32
*P. aeruginosa*	4	8	1	1

**Table 2:** Results of the Weibull model in 2 mL and 40 mL dissolution medium.

Parameter	40 mL Dissolution Volume	2 mL Dissolution Volume
*A*	100.2234	101.0138
*x_c_*	23.4049	9.7956
*d*	0.1593	0.9147
*k*	0.0008	165.4082

## Data Availability

The data presented in this study are available on request from the corresponding author. (The data are not publicly available due to privacy or ethical restrictions.)
